# Investigating the Road to Equity: A Scoping Review of Solutions to Mitigate Implicit Bias in Assessment within Medical Education

**DOI:** 10.5334/pme.1716

**Published:** 2025-03-03

**Authors:** Kristin E. Mangalindan, Tasha R. Wyatt, Kirsten R. Brown, Marina Shapiro, Lauren A. Maggio

**Affiliations:** 1 Uniformed Services University of Health Sciences and Internal Medicine physician, Naval Medical Center San Diego, San Diego, CA, US; 2 Department of Health Professions Education, Center for Health Professions Education, Uniformed Services University of Health Sciences, Bethesda, MD, US; 3 Department of Health Professions Education, Uniformed Services University of the Health Sciences, Bethesda, Maryland, US; 4 Department of Medical Education, University Illinois College of Medicine at Chicago, Chicago, IL, US

## Abstract

**Introduction:**

In medical education, assessments have high-stakes implications. Yet, assessments are rife with unconscious bias, which contributes to inequitable social structures. Implicit bias in assessment must be addressed because medical educators use assessments to guide learning and promote development of physicians’ careers. In this scoping review, the authors map the literature on implicit bias in assessment, as it applies to: 1) the types of implicit bias addressed, 2) the targets and types of interventions studied or proposed, and 3) how publications describe intervention efficacy.

**Methods:**

The authors conducted a scoping review of the literature on interventions to mitigate implicit bias that was published between January 2010 and August 2023. Author pairs independently screened articles for inclusion and extracted data. Discrepancies were resolved with discussion and consensus. Qualitative and quantitative analysis was informed by Anderson et al’s three assessment orientations: fairness, assessment for inclusion (AfI), and justice.

**Results:**

7,831 articles were identified; 54 articles were included. The majority (n = 37; 69%) of articles focus on implicit bias toward those underrepresented in medicine. Interventions to mitigate implicit bias were targeted toward admissions and applications, faculty training, recruitment, summative assessments, and evaluation templates. Interventions had *fairness* (n = 43; 96%) and *AfI* (n = 22; 49%) orientations; no articles used a *justice-orientation*. For the sub-set of research studies (n = 40), almost all (n = 34; 85%) examined a single program/institution.

**Discussion:**

This scoping review showed that more work is necessary to address different types of implicit biases, move scholarship beyond single-institution studies, refine existing interventions, and evaluate how efficacy is defined.

Medical educators have made concerted efforts to improve workforce diversity; however, inequitable social structures and bias persist [1]. Assessments (e.g. summative evaluations, interviews, applications) are one area where medical education establishes and perpetuates inequity. Although assessments are often viewed as neutral, drawing on the idea that they are objective measures, assessments in academic medicine are rife with unconscious bias and lead to harmful discrimination [2]. Implicit bias (also known as unconscious bias) refers to social stereotypes about groups of people that individuals form outside of their own conscious awareness [2]. Addressing implicit bias is imperative because medical educators use assessments to guide learning and promote career development.

Assessments are high stakes with the capacity to change an individual’s career trajectory. For example, the content of narrative evaluations for physicians underrepresented in medicine (UIM) and women physicians refers to personal attributes more than competency-related behaviors, raising concerns about evaluators’ ingrained implicit bias [3]. Further, in Program Director Letters of Recommendation, women and UIM were described in communal terms, reflecting their interpersonal skills and empathic behaviors, whereas White men were described with agency, reflecting behaviors of leadership and confidence [4]. Even slight differences in evaluations, like those that ascribe competency or agency to non-UIM physicians, can trigger an “amplification cascade” and stifle future opportunities [5]. Thus, it is unsurprising that White medical students are 5 times more likely than Black students to be honor society members [6]; women are only half as likely to reach senior leadership roles in academic medicine compared to men [7]; and only 8% of medical faculty in the United States (US) identify as UIM [8]. These studies suggest that inequity hides deep in the curriculum, masking as everyday educational activities that are rarely questioned [5,6,7,8].

Equity in assessment is a social construct; and as such equity holds multiple definitions or understandings [9]. In this study, we examine equity in assessment based on Anderson et al’s [10] three orientations–fairness, assessment for inclusion (AfI), and justice. *Fairness-oriented* assessment asserts that all learners should be afforded equal opportunities to be assessed using unbiased assessments; *AfI* emphasizes inclusion of all learners in assessment by minimizing discriminatory practices, rather than only addressing forces of partiality; and *justice-oriented* assessment argues that no assessment can be truly impartial because they will always reflect histories, sources of knowledge, and social and cultural characteristics and, thus, should be co-designed by marginalized learners. These three orientations are integral to the process and outcomes of assessment, including how assessment is conducted – its design, context, and use – and the equity stemming from the impacts of an assessment system, including decisions made from assessment data. While addressing implicit bias is critical and necessary to advance equity in assessment, we acknowledge that it is only one factor among many, like social accountability and culturally responsive assessments. We chose this framework so readers could identify and examine published interventions that align with their own orientation, consider expanding their efforts to include other orientations, and explore why some orientations are used more than others.

While medical education acknowledges implicit bias in assessment [2,3,4,6,7], at present, there is no published knowledge synthesis on solutions to mitigate its existence in all aspects of medical education. Recent reviews by Ismaeel et al [11] characterized implicit bias in observed assessments while Gottlieb et al [12] focused on holistic reviews – which can be components of bias mitigation in assessment, but can also be conducted to produce a workforce reflective of the population institutions serve. Therefore, the current study maps the literature on implicit bias in assessment, as it applies to: 1) the forms of implicit bias addressed, 2) the targets and types of interventions studied or proposed, and 3) how publications describe intervention efficacy. Our goal is to provide educators and leaders with an overview of potential interventions to mitigate implicit bias, in hopes of coming one step closer to realizing equity in academic medicine.

## Methods

We conducted a scoping review to synthesize and consolidate existing literature addressing mitigating implicit bias in assessments within medical education. Our review was guided by the six steps described by Arksey and O’Malley [13] and updated by Levac [14]. To report our review, we adhered to the PRISMA-ScR [15].

### Identifying the Research Question

We developed our research question based on a preliminary review of the literature. Our backgrounds also influenced the research question and overall conduct of the review. Our team consisted of 5 health professions education (HPE) researchers based in the US: KM is a second-generation Filipino American woman and clinician-educator interested in actionable change to address implicit bias within medicine and medical education; MS is a White woman HPE researcher interested in the intersection of health disparities, equity gaps, and bias; KB is a queer, disabled, White woman whose research examines the intersection of bias and disability; TW is a medical education researcher, White woman, interested in the intersection of bias and race/ethnicity; and LM is a White woman with a background in information science and expertise in conducting scoping reviews. Our collective experiences lead us to ask questions about what and how; the existing literature pointed us towards type of bias, interventions, and efficacy.

### Identifying Relevant Studies

A medical librarian, in close consultation with our team, searched PubMed, Web of Science, Embase, and ERIC databases on August 23, 2023. The search strategies, optimized for each database, incorporated combinations of keywords and controlled vocabulary terms representing the key concepts of implicit bias, medical education, and assessment. Terms included, but were not limited to: bias, equity, underrepresented in medicine, and racism. Medical education search criteria included: medical students, clerkship, residents, and faculty. Assessment terms included: educational measurement, evaluation, letter of recommendation, and interview. For complete search terms, see Appendix A. All citations were uploaded to Covidence, a knowledge synthesis tool for data management.

### Selecting Studies

Our literature search was limited to articles published after 2010, when the Association of American Medical Colleges (AAMC) announced an initiative on achieving health equity through inclusion [16]. Because implicit bias is socially situated – a construct of power that is grounded in historical, cultural, geographic, and political contexts [17] – we focused our review on research conducted in North America. To be included, studies needed to have or propose interventions in mitigating implicit bias in medical education. We excluded studies focused on mitigating implicit bias for patient care and clinical decision making, which is important, but outside this study’s scope. We also excluded interventions for fields outside of medical education (e.g. pharmacy).

Two investigators independently screened the article’s titles and abstracts for eligibility. The first author reviewed all articles; the others served as second reviewers. We met biweekly for one year to clarify ambiguities and resolve discordance in eligibility determination. We repeated the process for the full-text screening in which two authors independently read full text articles that met the initial inclusion criteria; disagreements were resolved via group consensus.

### Charting the Data

Before extracting the data, we piloted and refined a data charting tool for extraction. Extracted data included: publication type, year, geographic location, and medical specialty. We noted the type(s) of implicit bias addressed and separated interventions into level: individual, interpersonal, program, institutional, or policy. We then further subcategorized interventions into targets and types. If the article was a study, we noted how efficacy was measured and if the intervention(s) was/were effective at mitigating implicit bias. We also extracted: whether the first author self-identified as being a member of the group addressed in the article, the geographic location and institutional affiliation of the first author, whether a theoretical framework was used, and what the article noted as limitations and future directions. We included this information to better understand the circumstances and positionality with which the researchers approached solutions to mitigate implicit bias.

### Collating, Summarizing, and Reporting the Results

We consolidated and synthesized the data, analyzing it with quantitative and qualitative lenses. Quantitatively, we described the corpus to include the kind of publication, year, type of intervention, etc. to provide an overview of the articles’ nature, extent, and distribution. Qualitatively, we reviewed the data pertaining to our research questions and identified the current state of knowledge on bias mitigation in medical education. KM and KB used descriptive, In Vivo, and process techniques [18] to code the data and then engaged in thematic analysis [19,20]. Throughout the process, we used multiple coders to address coding reliability and created an interpretive codebook to maintain a shared understanding among authors. We also engaged in ongoing reflexivity via memoing – recording our insights and interpretations of the data throughout the research process – and discussed our positionality and varied experiences with implicit bias during team meetings.

Once the data were organized and analyzed, we classified the interventions into Anderson et al’s three orientations. For instance, one study that attempted to address equity in Honor Medical Society selections described expanding eligibility criteria from >50% honors grades in core clerkships to >25% honors grades in core clerkships with a goal to increase eligibility and selection outcomes for UIM vs non-UIM students [21]. Given the goal of the intervention, this article was labeled as “*Assessment for Inclusion (AfI)*” because it targeted equity through inclusive practices.

### Consultation

We shared our results with 3 experts (including Graduate Medical Education leaders and diversity, equity, and inclusion experts) via email to check the relevance of our findings with their experience. While overall the findings resonated with the stakeholders, we incorporated minor edits to delineate assessment definitions and added a discussion of the Computer-Based Assessment for Sampling Personality Characteristics (CASPer); a situational judgment test to measure noncognitive skills as a component of holistic review [22,23].

## Results

**Figure 1:**
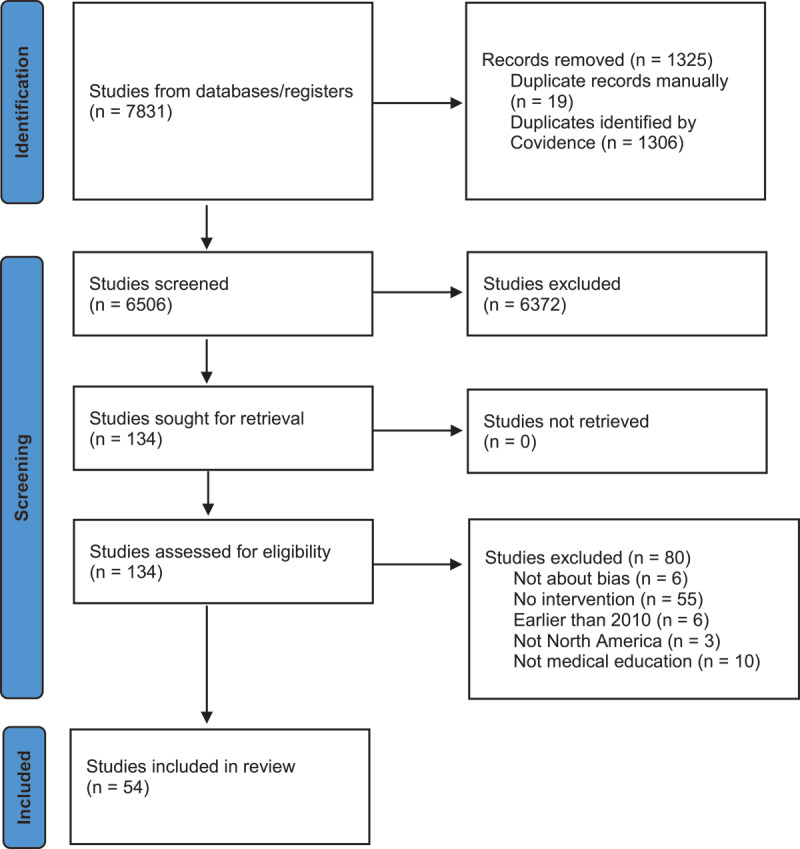
PRISMA Flow Diagram.

We identified 7,831 publications, of which 54 articles were included in the final analysis [24,25,26,27,28,29,30,31,32,33,34,35,36,37,38,39,40,41,42,43,44,45,46,47,48,49,50,51,52,53,54,55,56,57,58]; See Figure 1 for PRISMA flow diagram. We first present the overall characteristics of the included articles and then address each research question in relation to Anderson et al’s orientations [10].

**Figure 2:**
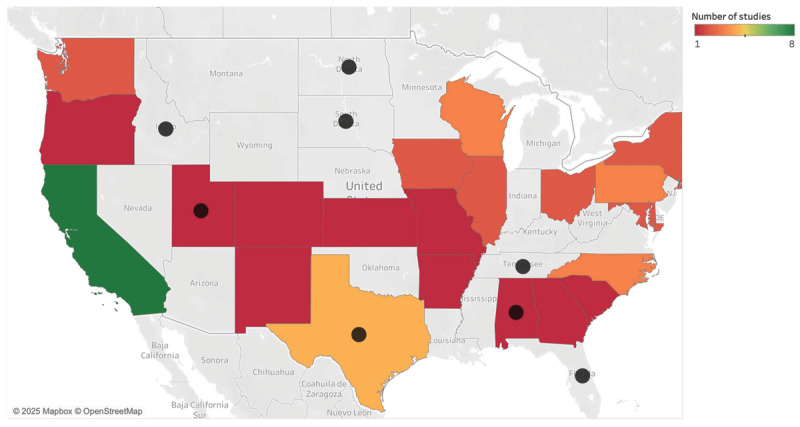
Heat map of first author’s institutional affiliation superimposed with current anti-bias legislation. Colors show the sum of studies conducted in that state. The black circles indicate states with anti-bias legislation.

Most articles were published in 2019 or later (see Table 1), with first authors primarily based in the US across 19 states (see Figure 2). Articles were published in 35 different journals; *Academic Medicin*e (n = 10; 19%) [25,28,31,35,40,45,52,58,59,60] and the *Journal of Surgical Education* (n = 5; 9%) [39,44,53,55,61,62,63] were the most prevalent. Eight articles used theoretical frameworks, which included critical consciousness [59], Dewey’s theoretical notion of reflective action [64], and a discussion on postpositivist, constructivist, and critical theories [52].

**Table 1:** Characteristics of articles identified in a scoping review of interventions to mitigate bias in medical education, 2010–2023.


DOMAIN	FEATURE	NO. (%) OF ARTICLES (N = 54)

**Publication Year**	2019 or later	49 (91%)

**Publication Type**	Studies	40 (75%)

	Scholarly Perspectives	9 (17%)

	Innovations	3 (6%)

	Reviews	2 (4%)

**Location of First Author**	United States	49 (91%)

	Canada	4 (7%)

	United Kingdom	1 (2%)

**Target Population**	UME	13 (24%)

	GME	32 (59%)

	CME	3 (6%)

	Combination	6 (11%)

**GME Specialties**	General Surgery	5 (9%)

	Otolaryngology	3 (6%)

	Family Medicine	1 (2%)

	Internal Medicine	1 (2%)

	Orthopedic Surgery	1 (2%)

**Theoretical Framework**	Yes	8 (15%)

**First Author Self-Identification**	Yes	2 (4%)


From the subset of research studies (n = 40), almost all (n = 34; 85%) examined a single program or individual institution. The average number of participants was 872 (SD = 1573, Range = 5–8243). The largest study, which had 8243 participants, examined a Machine Learning Decision Support Tool to screen admissions applications to mitigate bias [31].

### What forms of implicit bias are being addressed?

Anti-bias interventions were proposed and/or designed to minimize bias for different populations of marginalized learners. The majority (n = 37; 69%) targeted UIM individuals, who experience racial disparities and implicit bias throughout their education and in traditional residency selection metrics [65,66]. Implicit gender bias was the next most common (n = 22; 41%). This was often described in relation to gender-based differences in the traits ascribed to trainees and highlighted when performing roles as a leader, manager, or within a team [67]. Articles often addressed multiple groups of marginalized learners (i.e.: UIM and women), but not the intersection of both identities. Thirteen articles (24%) used broad phrases, such as “unseen bias” [31] or “bias in the residency application process [30].” The least common was socioeconomic disadvantage (n = 7; 13%), in which authors argued that when implicit bias is coupled with lack of equal opportunities due to structural barriers [68], the disadvantaged experience poorer academic outcomes [69].

### What areas of medical education were targeted and what types of interventions were studied or proposed?

Eighty percent (n = 43) described the implementation of interventions and reported outcomes. Eleven articles (20%) featured interventions or potential strategies for mitigating bias. For example, a perspective article proposed developing institutional procedures to evaluate assessments [26] while another discussed the potential of implementing mandatory anti-bias training for evaluators and establishing clear criteria for competency-based assessments [70]. Of the articles that implemented an intervention, 30 (70%) occurred at the program level, 11 (26%) at the institutional level, and two (5%) at both the interpersonal and individual levels [30,71].

We categorized the interventions using Anderson et al.’s equity assessment orientations of *fairness* (n = 43; 96%), *AfI* (n = 22; 49%), and *justice* (n = 0; 0%), noting that some articles overlapped orientations. Authors that were *fairness-oriented* adopted practices and strategies to increase quality/validity of assessment tools, control the quality of group decision-making, and improve assessor expertise/agility. *AfI* primarily focused on implementing tools for shared decision making instead of co-designing flexible, individualized assessment systems, which is the goal of inclusion equity. There were no studies where a structural approach was taken to acknowledge differences in performance (i.e. *justice-orientation*).

Anti-bias interventions targeted six areas of medical education. We describe interventions within each category of implementation below (see Table 2).

**Table 2:** Summary of intervention targets and types identified in a scoping review of interventions to mitigate bias in medical education, 2010–2023.


INTERVENTION TARGET	INTERVENTION TYPE	DESCRIPTION	NO. (%) OF ARTICLES (N = 54)

**Admissions and Applications**	Integrating Core Values	Integrating an organization’s core values into the assessment process	12 (23%)

	Diversifying Faculty	Incorporating diverse faculty evaluators into the application process	14 (26%)

	Adding Questions to Application	Adding questions about life experience or reflection questions based on the organization’s core values	5 (9%)

	Expansion of Eligibility Criteria	Removing test score criteria, liberalizing score/grade filters	7 (13%)

	Holistic Review	Deemphasizing academic credentials	7 (13%)

	Screening Algorithms	Designing computer algorithms to screen applications	4 (7%)

	Blinding Application Components	Blinding evaluators to pictures, names, transcripts, or test scores	13 (24%)

	Independent Evaluators	2–6 independent reviewers review applications	7 (13%)

	Committee Reviews	Committee decides on advancement/rank lists	5 (9%)

	Scoring Systems	Structured and/or weighted scoring system according to program needs	12 (23%)

	Training screeners/interviewers	Training to walk through scoring rubrics and grade sample applicants	5 (9%)

**Interviews**	Standardized Interview Questions	Standardized interview questions across all applicants	8 (15%)

	Behavioral-Based Interview Questions	Center questions on past behaviors or situational questions based on domains valued by the program	4 (7%)

**Recruitment**	Creating a DEI committee/position	Goal to increase diversity within programs/institutions	5 (9%)

	Interactions with UIM trainees/faculty	Intentional interactions with UIM trainees/faculty	4 (7%)

	Expanding the Pipeline	Creating programs for UIM learners from college to medical school or medical school to residency	5 (9%)

**Faculty Training**	Implicit Bias Training	Mandatory implicit bias training for evaluators	19 (35%)

	Faculty Development	Workshops or curricula aimed at mitigating bias in assessment	8 (15%)

**Summative Assessments**	Competency-Based Assessments	Evaluators assess learner progress based on observable behaviors	3 (6%)

	Internal Validation of Assessments	Creating institutional procedures to validate reliability and utility of assessments	3 (6%)

**Evaluation Templates**	Standardization of Evaluation Letters	Structured templates to identify components important to reviewing committees	2 (4%)


#### Admissions

Thirty (67%) interventions targeted admissions and applications to medical school, residency, fellowship, or honors societies. In this category, articles integrated the organization’s core values into the assessment process (n = 12, 23%) and ensured faculty assessing trainees were diverse (n = 14, 26%) in gender, ethnicity, background, interests, specialty, and teaching experience. In relation to Anderson et al’s orientations, these interventions approach a *justice-orientation*, but because the faculty did not co-design the assessment system with key stakeholders, or ensure the organization’s core values took historical, social, cultural, and political context into account, they were not included in this orientation.

We characterized 25 (46%) studies with a *fairness-orientation*. These interventions were geared toward mitigating implicit bias through a variety of approaches. For example, there were efforts to blind application components so reviewers are not biased by names, appearances, or academic credentials [21,25,32,34,38,50,55,61,72,73,74,75,76]. Independent evaluators were used to mitigate bias by individuals in the screening process [25,28,32,50,62,76,77]. Scoring systems were created to meet specific program needs (e.g., geographic location, academic performance, and cultural competence) [21,25,29,33,37,41,44,50,62,63,72,73].

Twenty (37%) articles were categorized as *AfI*. Interventions in this category included adding questions to applications, ranging from “Distance Traveled” (a metaphorical distance that an applicant travels throughout life and composite measure of achievement), life experience, and reflection questions based on the organization’s core values [21,29,42,50,73]. By integrating reflective questions into applications, marginalized learners are given an opportunity to capture the unspoken accomplishments in their academic career that may not be captured by traditional metrics (e.g., publications). Other articles expanded eligibility criteria [21,43,61,72,73,74,76] as a means of closing the achievement gap between UIM and non-UIM learners [78].

Interviews (n = 14; 31%) were a large subset of the admissions and applications interventions. All interventions geared toward interviews approached equity in the *fairness-orientation* category. This included using a structured interview process, with standardized questions to improve reliability, validity, and fairness [28,33,39,43,44,48,61,74,75]; using questions centered around past behaviors and situational questions to assess the applicant’s likelihood of success based on attributes valued by the program [43,48,56,77]; and training screeners and interviewers on scoring rubrics [27,33,43,44,56]. No interventions in the interview process were targeted toward *AfI*.

#### Recruitment

Recruitment refers to processes in place to increase applications to a program or institution. Nine (20%) articles addressed recruitment as an intervention to mitigate bias [35,37,43,47,50,72,75,76,79]. While recruitment to medical schools, residencies, or fellowships is not traditionally considered a form of assessment, it does approach process equity (i.e.: equity of the learning environment in which assessment is conducted) from an *AfI* perspective. Recruitment interventions included creating DEI positions/committees, fostering applicants’ interactions with UIM trainees/faculty, and expanding the college to residency pipelines for UIM learners.

#### Faculty Training

Faculty training was common (n = 27; 60%), with 19 articles discussing implicit bias training and eight focused on faculty development workshops or curricula. Faculty training ranged from 20–120 minute workshops. Trainings were focused on bias mitigation so we categorized these interventions under *fairness-orientation*. Implicit bias training was used as a general concept and not clearly delineated in six articles. Training was accompanied by the Implicit Association Test in five (19%) articles [33,37,39,51,80]. Specific implicit bias training mentioned included the AAMC’s “Everyday Bias for Healthcare Professionals,” [72] AAMC’s “Bias Breakers” workshop [64], and National Research Mentoring Network’s Unconscious Bias Course [49]. Two articles discussed guides or best practices for evaluators to review prior to or during their assessments [26,36]. These guides included principles like finding common identity formation, enacting stereotype negation, and considering sources of bias. The only *AfI* article described a collection of workshops to obtain a Teaching for Equity and Inclusion Certificate [59], which included workshops on Holistic Approach to Selection and Hiring and Inclusive Leadership.

One qualitative study approached equity on assessment from a *justice-orientation*, but did not co-design the assessment system with key stakeholders [54]. This study focused on mandatory faculty training, included historical underpinnings of anti-Black racism, drew on outside expertise, and discussed the importance of resource allocation and structural change.

#### Summative Assessments

Summative assessments (n = 8; 18%), such as end-of-rotation assessments, Clinical Competency Committee assessments, or assessments for promotion were all based on the *fairness-orientation*. Common patterns included using direct observation, competency-based assessments, and multiple data points, developing institutional procedures to internally validate the reliability and utility of assessments, and incorporating this information into annual reviews.

#### Evaluation Templates

Two articles (4%) discussed standardization of evaluation letters, with one focusing on Medical Student Performance Evaluations (MSPEs) and the other on faculty promotion letters. Templates were structured such that the writer identified components of a resume that were significant to a reviewing committee. Both evaluation templates approached equity in assessment from a *fairness-orientation*, ensuring that information was competency-based, comprehensive, and effective in lieu of focusing on personal attributes.

### How did authors describe the efficacy of interventions?

Most studies (85%) examined a single program or institution; therefore, descriptions of efficacy were context-bound. Below, we map how authors characterized efficacy in the 43 studies that reported outcomes. We did not assess the study’s efficacy; that type of analysis is typically outside the bounds of a scoping review.

Frequently (n = 22; 51%), efficacy was measured by comparing the percentage of marginalized learners selected for interviews, admissions, and residency/fellowship programs in terms of pre- and post-intervention(s). This approach aligns with the *fairness-orientation*, particularly around tracking outcome equity.

Fifteen (35%) studies framed efficacy in terms of increasing the number of marginalized learners at specific points of interaction (e.g., interview invitations, admission, and/or matches). All of these studies were multipronged, incorporating variations of: diverse evaluating faculty, expanding test score eligibility, qualitative test scores, incorporating additional questions, independent reviewers, increased recruitment, masking interviewers to the applicant’s academic record, and using standardized interview questions [21,27,29,46,50,55,57,61,72,73,75,76,77,79].

Nine (21%) studies measured the efficacy of interventions with surveys on knowledge, skills, or attitude changes before and after the intervention. The majority of these studies were faculty trainings; survey questions asked about training satisfaction, knowledge and skills around bias recognition and mitigation, pre- and post- Implicit Association Tests (IATs) [30,45,51,59,60,64,80]. Other studies measured perspectives on the blinded interview process [38] and on subjective utility of an added question to an application [42]. No studies in this category measured change or impact on evaluations, promotion, or advancement for marginalized learners.

Seven (16%) studies did not achieve the goal of mitigating implicit bias in assessment. Two studies were algorithms that applied specific scores and weights to application components [62,63]. One study masked MCAT scores during the admissions process [25], one masked name and photographs during the screening processes [32], and one masked interviewers to test scores, transcripts, and LORs [34].

One (2%) study had incongruent efficacy for gender and UIM bias, demonstrating that behavioral-based interviews mitigated implicit bias for UIM bias, but not for gender bias [56].

## Discussion

While there is increasing attention to interventions to mitigate bias in assessment, our scoping review revealed that there is more work to be done regarding the types of implicit biases addressed, the refinement of existing interventions to mitigate it, exploration of new interventions from different orientations, and re-examining how to study intervention efficacy.

### Types of Bias Addressed

Several studies sought to mitigate bias for UIM, gender, and/or socioeconomic disadvantage or often addressed multiple groups of marginalized learners (ie: UIM and women). Yet none addressed the intersection of these biases – overlooking that some learners have unique contexts necessitating interventions that target intersectionality. Further, almost a quarter of the articles did not specify what type of bias they were trying to mitigate, positioning bias as nonspecific and uniform. Understanding the origins and effects of biases is integral to modifying or creating assessment systems. Confirming recent reviews [11,12]; ableism, heterosexism, and religion bias were minimally studied.

### Targets and Types of Interventions

Interventions to mitigate bias in assessment were focused on the orientations of *fairness* and *AfI*, which do not fundamentally acknowledge, incorporate, or shift the structures in society that created bias. No interventions targeted a *justice-orientation*, which would mitigate bias through co-designed assessment systems with community-led and community-controlled decision-making, acknowledging that what is assessed is frequently designed with the more typical student in mind, those privileged by societal structures. We theorize that this is because it is difficult to dismantle current assessment systems. Rethinking assessment systems for justice is time intensive and would require many stakeholders at multiple points in the system, not the least of which would be regulatory bodies. However, a *justice-orientation* would explicitly address the root causes of implicit bias and acknowledge that assessment cannot be truly impartial. Further, it would empower marginalized populations to collaborate on assessment systems to bring the medical education community one step closer to equity.

Implicit bias exists because of systems and structures in place that have disproportionately disadvantaged marginalized learners for centuries. Interventions targeting *fairness* or *AfI* orientations, like masking academic records, names, and photographs or incorporating CASPer scores as a surrogate for noncognitive skills in the admissions screening process, only skim the surface. Marginalized learners are disadvantaged at multiple levels in the pre-application process beyond test scores, GPAs, and clerkship grades. This includes the ability to engage in extracurricular activities, volunteer work, leadership positions, and scholarly activity. Interventions are being developed outside of the communities that they affect and, instead, need to be co-developed with these learners.

### Efficacy of Interventions

The strategies and interventions implemented were frequently effective in bias mitigation for marginalized learners when used in tandem more as a program of assessment towards equity, rather than a single effort [21,27,29,46,47,50,55,57,61,72,73,75,76,77,79]. However, researchers’ multipronged approaches made it difficult to assess the most effective anti-bias intervention and provided limited evidence as to what worked and why.

A similar problem occurred with faculty training, which was a common intervention. The efficacy of faculty training was measured with pre/post-intervention surveys [30,45,49,51,59,60,64], reflecting short-term changes, but there were no longitudinal studies that measured whether faculty training resulted in lasting change or improvement in outcome equity. By focusing only on the intervention itself, researchers miss a developmental perspective that would enable permanent shifts or change. Similarly, mandatory implicit bias training was frequently mentioned as an intervention [27,33,37,38,39,45,47,49,50,51,54,55,58,59,64,70,72,73,80], but limited details were discussed regarding its content or quality, leaving readers with little guidance on how to implement such efforts.

Finally, efficacy was often studied with a *fairness-orientation*, particularly around tracking outcome equity (i.e. percentage of marginalized learners that applied and those selected). Such an approach is meant to examine how populations increase as a result of the intervention but is a weak form of analysis because it does not attend to nuances in outcomes. For example, although the number of Black individuals may have been advanced or selected for a program using a particular intervention, merely measuring the number of Black residents pre/post intervention tells one nothing about whether these individuals were immigrants from the Caribbean or Sub-Saharan Africa or are descended from slavery, and have therefore experienced generations of racism, discrimination, and educational barriers.

## Limitations

Our findings must be considered in light of the study’s limitations. While our search strategy was robust, we may have inadvertently missed articles that characterized implicit bias in assessment with different terminology (i.e.: impartiality, transparency). Our focus on North America excluded insights from other world regions, and future research should consider including multiple continents. Research does not occur in a sociopolitical vacuum. Most of the studies included in this review were conducted in California, which has strong anti-discrimination statutes around equal educational opportunities [81]. Whereas, other states that developed interventions were in Texas, Utah, and Alabama, which now have restrictive policies around DEI efforts [82]. The Supreme Court limited the use of affirmative action in higher education admissions [83]. Researchers should be attentive to these larger conversations as they analyze and interpret anti-bias interventions, especially around what can legally be studied, or not studied, in various socio-political contexts.

## Conclusion

This scoping review maps the landscape of interventions targeting bias in assessment, and highlights that there is much work to be done in creating actual change to assessment practices. Until medical education recognizes that achieving equity is a larger, structural issue in a vast medical education ecosystem in which target populations are highly diverse in terms of background experiences and identities and assessment is highly complex, we will not achieve equity. Future research should focus on understanding the origins and effects of different types (and intersections) of implicit bias, examining individual anti-bias interventions for efficacy (and reasons for efficacy), long-term effects of faculty training on bias mitigation, and, finally, initiating development of bias mitigation strategies from a *justice-orientation*.

## Appendix A

**Table:**

DATABASE	SEARCH PARAMETERS

PubMed Search Strategy (n = 2,355) Limits: 1995-Present NOT: letter, comment, or editorial (n = 2,132)	( (“Education, medical”[mh] OR “students, medical”[mh] OR “medical student”[title/abstract:~2] OR “medical students”[title/abstract:~2] OR learner[tiab] OR learners[tiab] OR “medical school”[tiab] OR “medical schools”[tiab] OR clerkship[tiab] OR residency[tiab] OR resident* OR faculty[tiab] OR “underrepresented in medicine” OR “Underrepresented Minorities”[title/abstract:~3] OR Underrepresented[ti] OR “underrepresented minority”[title/abstract:~2]) AND (“Educational measurement”[mh] OR “formative feedback”[mh] OR “healthcare education”[tiab] OR “assessment learners”[title/abstract:~4] OR “assessment education”[title/abstract:~2] OR “clinical assessment”[title/abstract:~2] OR “medical education assessment” OR “Medical Student Performance Evaluation” OR “Dean’s Letter” OR interview* OR “letters of recommendation” OR “letter of recommendation”) AND ((Bias OR prejudice OR racism OR equity OR homophobia OR Ageism OR sexism OR prejudice[mh] OR prejudice) OR “mitigating bias” OR (diversity AND differences AND increas*)) ) NOT (newborn[tiab] OR infant[tiab] OR child[tiab] OR adolescent[tiab] OR nursing[mh] OR “education, nursing”[mh] OR nursing[ti] OR nurses[ti] OR dentists[mh] OR dentistry[ti] OR pharmacists[mh] OR diagnosis[ti] OR patient[ti] OR patients[ti] OR “Diseases Category”[Mesh] OR protocol[ti] OR covid) OR Letter[Publication Type] OR Comment[Publication Type] OR Editorial[Publication Type])

Embase Search Strategy (n = 1,799) Limits: *1995-Present *Article OR Review publication type (n = 1,237)	‘medical education’/exp OR ‘medical education’ OR ‘medical students’/exp OR learner:ab,ti OR learners:ab,ti OR “medical school”:ab,ti OR “medical schools”:ab,ti OR clerkship:ab,ti OR residency:ab,ti OR resident* OR faculty:ab,ti OR “underrepresented in medicine” OR underrepresented:ti (medical NEAR/2 (student OR students)) OR (underrepresented NEAR/2 minorities) OR (underrepresented NEAR/2 minority) 1 OR 2 ‘education’/exp OR “educational measurement”:ab,ti OR ‘constructive feedback’:ab,ti OR “formative feedback” OR “healthcare education”:ab,ti OR “clinical assessment” OR “medical student performance evaluation” OR “dean’s letter” OR interview* OR “letters of recommendation” OR “letter of recommendation” (assessment NEAR/4 learners) OR (assessment NEAR/2 education) 4 OR 5 Bias OR prejudice OR racism OR equity OR homophobia OR ageism OR sexism OR ‘prejudice’/exp OR prejudice OR “mitigating bias” OR (diversity AND differences AND increas*) 3 AND 6 AND 7 ‘juvenile’/exp OR ‘nursing’/exp OR ‘nursing education’/exp OR nursing:ti OR nurses:ti OR ‘dentist’/exp OR dentistry:ti OR ‘paramedical personnel’/exp OR diagnosis:ti OR patient:ti OR patients:ti OR ‘diseases’/exp OR protocol:ti OR covid:ti OR ‘procedures, parameters and devices’/exp 8 NOT 9

Web of Science (n = 3,452) Limits: 1995-Present Article OR Review (n = 2,780)	TS = (Medical NEAR/2 Education) OR TS = (medical NEAR/2 student*) OR TS = (residency) OR TS = (resident*) OR TS = (faculty) OR TS = (“underrepresented in medicine”) OR TS = (underrepresented NEAR/2 minorities) OR TI = (underrepresented) OR TS = (underrepresented NEAR/2 minority) TS = (education OR “educational measurement” OR “constructive feedback” OR “formative feedback” OR “healthcare education” OR “clinical assessment” “medical student performance evaluation” OR “dean’s letter” OR interview* OR “letters of recommendation” OR “letter of recommendation” OR assessment NEAR/2 learners OR assessment NEAR/2 education) TS = (Bias OR prejudice OR racism OR equity OR homophobia OR Ageism OR sexism OR prejudice OR “mitigating bias”) OR TS = (diversity AND differences AND increas*) #1 AND #2 AND #3 TS = (newborn OR infant OR child OR adolescent OR nursing OR nurses OR dentists OR dentistry OR pharmacists OR covid) OR TI = (diagnosis OR patient* OR protocol) #4 NOT #5 #6 AND PY = (1995–2023)

PsychINFO (n = 2,415) Limits: 1995-Present Academic Journals (n = 1,682)	(medical education) OR (medical student*) OR learner* OR (medical school*) OR clerkship OR residency OR resident* OR faculty OR “underrepresented in medicine” OR “Underrepresented Minorities” OR TI Underrepresented OR “underrepresented minority” “Educational measurement” OR “formative feedback” OR “healthcare education” OR “assessment of learners” OR “clinical assessment” OR “education assessment” OR “medical education assessment” OR “Medical Student Performance Evaluation” OR “Dean’s Letter” OR interview* OR “letters of recommendation” OR “letter of recommendation” Bias OR prejudice OR racism OR equity OR homophobia OR Ageism OR sexism OR prejudice OR “mitigating bias” OR (diversity AND differences AND increas*) newborn OR infant OR child OR OR adolescent OR nursing OR nurses OR dentists OR dentistry OR pharmacists OR TI diagnosis OR TI patient OR TI patients OR TI protocol
